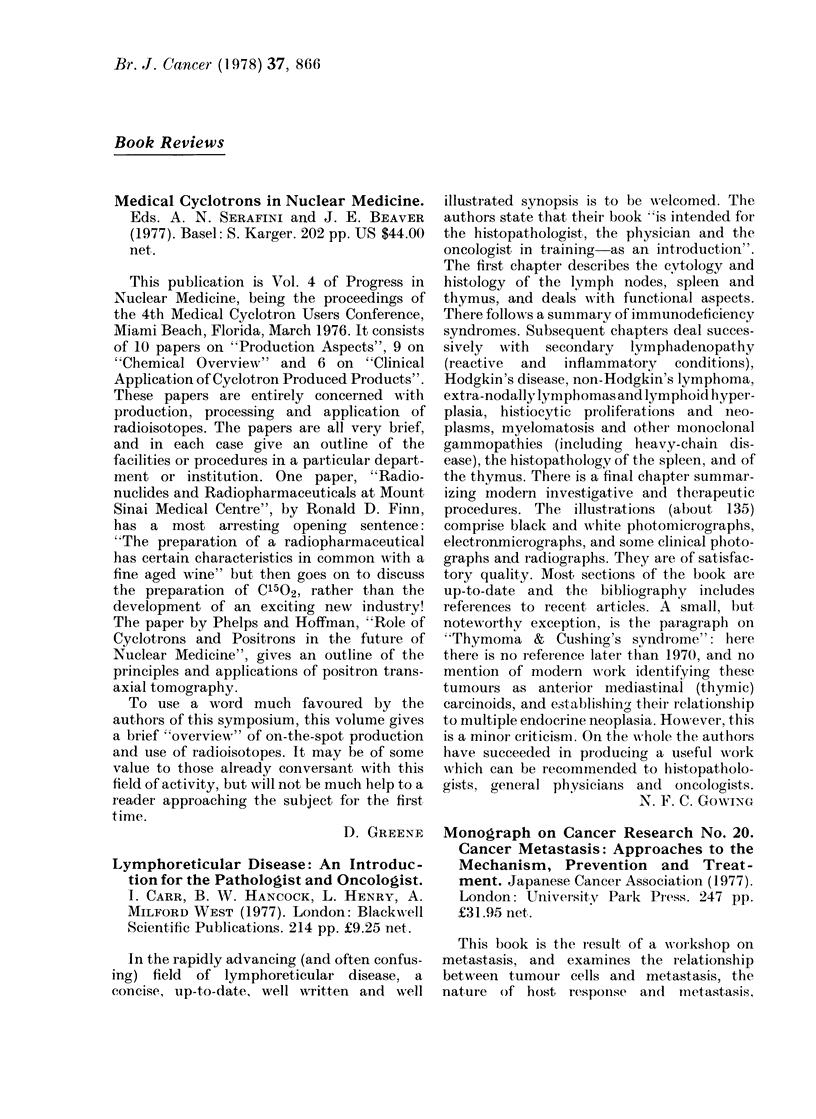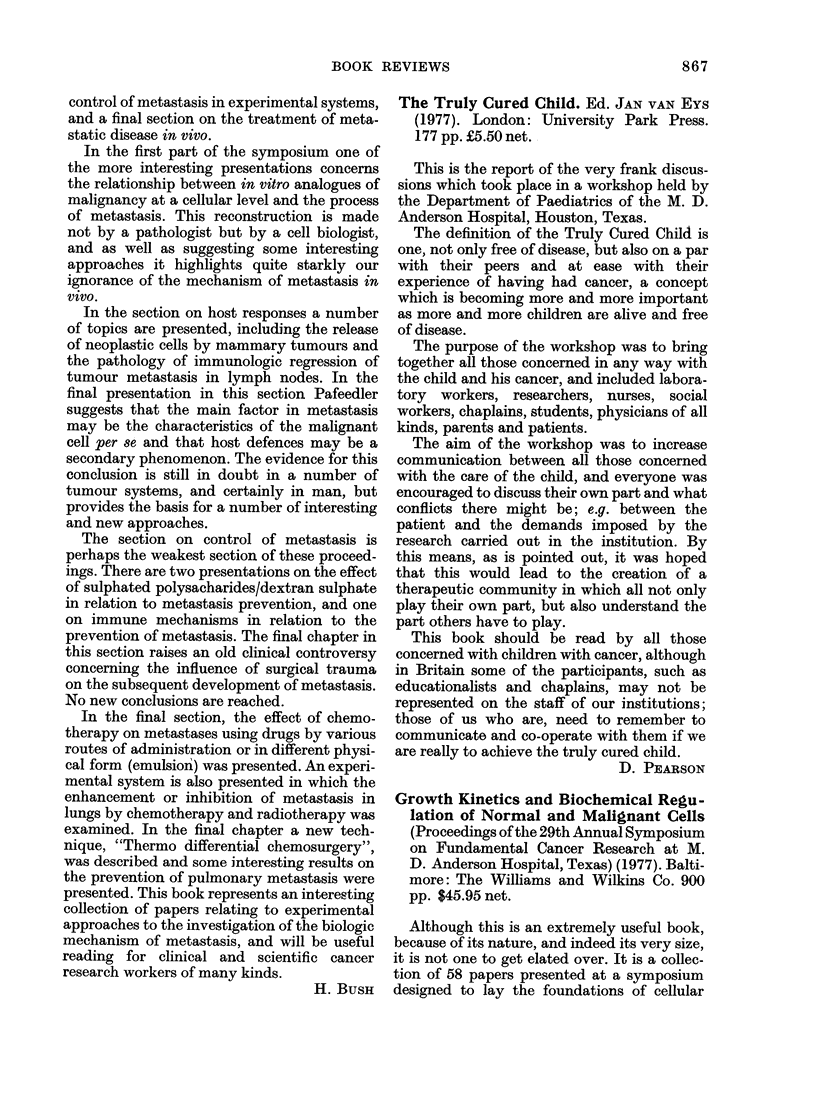# Monograph on Cancer Research No. 20. Cancer Metastasis: Approaches to the Mechanism, Prevention and Treatment

**Published:** 1978-05

**Authors:** H. Bush


**Monograph on Cancer Research No. 20. Cancer Metastasis: Approaches to the Mechanism, Prevention and Treatment.** Japanese Cancer Association (1977). London: University Park Press. 247 pp. £31.95 net.

This book is the result of a workshop on metastasis, and examines the relationship between tumour cells and metastasis, the nature of host response and metastasis, control of metastasis in experimental systems, and a final section on the treatment of metastatic disease *in vivo*.

In the first part of the symposium one of the more interesting presentations concerns the relationship between *in vitro* analogues of malignancy at a cellular level and the process of metastasis. This reconstruction is made not by a pathologist but by a cell biologist, and as well as suggesting some interesting approaches it highlights quite starkly our ignorance of the mechanism of metastasis *in vivo*.

In the section on host responses a number of topics are presented, including the release of neoplastic cells by mammary tumours and the pathology of immunologic regression of tumour metastasis in lymph nodes. In the final presentation in this section Pafeedler suggests that the main factor in metastasis may be the characteristics of the malignant cell *per se* and that host defences may be a secondary phenomenon. The evidence for this conclusion is still in doubt in a number of tumour systems, and certainly in man, but provides the basis for a number of interesting and new approaches.

The section on control of metastasis is perhaps the weakest section of these proceedings. There are two presentations on the effect of sulphated polysacharides/dextran sulphate in relation to metastasis prevention, and one on immune mechanisms in relation to the prevention of metastasis. The final chapter in this section raises an old clinical controversy concerning the influence of surgical trauma on the subsequent development of metastasis. No new conclusions are reached.

In the final section, the effect of chemotherapy on metastases using drugs by various routes of administration or in different physical form (emulsion) was presented. An experimental system is also presented in which the enhancement or inhibition of metastasis in lungs by chemotherapy and radiotherapy was examined. In the final chapter a new technique, "Thermo differential chemosurgery", was described and some interesting results on the prevention of pulmonary metastasis were presented. This book represents an interesting collection of papers relating to experimental approaches to the investigation of the biologic mechanism of metastasis, and will be useful reading for clinical and scientific cancer research workers of many kinds.

H. Bush